# Supplementary material for: Family Attachment, Sexuality, and Sexual Recidivism in a Sample of Italian Sexual Offenders: Preliminary Results
Source: Healthcare (Basel). 2023 May 29;11(11):1586. doi: 10.3390/healthcare11111586 (PMC10253209; doi:10.3390/healthcare11111586)
Supplement: Supplementary file 1 [file healthcare-11-01586-s001.zip › healthcare-2321275-supplementary.pdf]

Supplementary Materials

Compulsive Sexual Behavior Inventory (CSBI)

Adapted in Italian by the researchers of the University of Cassino and Southern Lazio

Table S1. Read the statements below and tick the number that most closely matches your degree of agreement. There are no right or wrong answers: 1 = Never 2 = Rarely 3 = Occasionally 4 = Frequently 5 = Very Frequently

1	Quanto spesso hai avuto difficoltà a controllare i tuoi impulsi sessuali?	1	2	3	4	5
2	Quando spesso ti sei sentito incapace di controllare il tuo comportamento sessuale?	1	2	3	4	5
3	Quanto spesso hai usato il sesso per risolvere problemi e preoccupazioni nella tua vita?	1	2	3	4	5
4	Quanto spesso ti sei sentito in colpa o ti sei vergognato di alcuni aspetti del tuo comportamento sessuale?	1	2	3	4	5
5	Quante volte hai tenuto nascosto o mascherato il tuo comportamento sessuale agli altri?	1	2	3	4	5
6	Quanto spesso ti sei sentito incapace di gestire i tuoi impulsi sessuali?	1	2	3	4	5
7	Quanto spesso hai preso l'impegno o hai promesso di cambiare il tuo comportamento sessuale?	1	2	3	4	5
8	Quanto spesso i tuoi pensieri o comportamenti sessuali hanno interferito nella costruzione di amicizie?	1	2	3	4	5
9	Quanto spesso hai inventato scuse e ragioni per giustificare il tuo comportamento sessuale?	1	2	3	4	5
10	Quanto spesso hai perso delle opportunità di attività produttive o di miglioramento a causa della tua attività sessuale?	1	2	3	4	5
11	Quanto spesso le tue attività sessuali ti hanno causato problemi finanziari?	1	2	3	4	5
12	Quanto spesso ti sei sentito emotivamente distante mentre facevi sesso con qualcuno?	1	2	3	4	5
13	Quanto spesso hai fatto sesso o ti sei masturbato più di quanto volessi?	1	2	3	4	5
14	Quanto spesso sei stato arrestato o sei stato tenuto in custodia per il tuo comportamento sessuale?	1	2	3	4	5
15	Hai forzato qualcuno contro la sua volontà a fare sesso?	1	2	3	4	5
16	Hai mai colpito, preso a calci, dato un pugno, schiaffeggiato, atterrato, soffocato, controllato, o picchiato qualcuno dei tuoi partner?	1	2	3	4	5
17	Hai provocato dolore fisico a qualcuno per il tuo piacere sessuale?	1	2	3	4	5
18	Durante una lite, sei stato colpito, preso a calci, preso a pugni, schiaffeggiato, atterrato, soffocato, controllato, o picchiato dal tuo attuale o più recente partner?	1	2	3	4	5
19	Hai ricevuto dolore fisico con lo scopo di avere piacere sessuale?	1	2	3	4	5
20	Hai ricevuto soldi per fare sesso?	1	2	3	4	5

21	Sei stato forzato a fare sesso con tuo marito, tua moglie o con il tuo amante?	1	2	3	4	5
22	Sei stato guardato mentre ti masturbavi o facevi sesso senza il tuo consenso?	1	2	3	4	5

Sexual Sensation Seeking Scale (SSSS)

Adapted in Italian by the researchers of the University of Cassino and Southern Lazio.

Table S2. Read the statements below and tick the number that most closely matches your degree of agreement. There are no right or wrong answers: 1 = Not at all Like Me 2 = Slightly Like Me 3 = Mainly Like Me4 = Very Much Like Me

1	Mi piacciono incontri sessuali selvaggi e disinibiti	1	2	3	4
2	Le sensazioni fisiche sono l'aspetto più importante del sesso	1	2	3	4
3	I miei partner sessuali probabilmente pensano che io sia uno che si assume dei rischi	1	2	3	4
4	Quando si tratta di sesso, per me l'attrazione sessuale è più importante di quanto bene conosca la persona	1	2	3	4
5	Mi piace stare con persone sensuali	1	2	3	4
6	Mi piare guardare video pornografici	1	2	3	4
7	Sono interessato a provare nuove esperienze sessuali	1	2	3	4
8	Mi sento come se stessi esplorando la mia sessualità	1	2	3	4
9	Mi piace provare nuove esperienze e sensazioni sessuali	1	2	3	4
10	Mi piace la sensazione di avere rapporti sessuali senza preservativo	1	2	3	4

High-Risk Situation Checklist

Adapted in Italian by the researchers of the University of Cassino and Southern Lazio.

Table S3. Read the following list of possible situations or conditions that could cause you to relapse into the behavior you would like to change and indicate the ones that best identify what you are feeling.

**Quali emozioni negative ti porterebbero ad una ricaduta?**		
N	1	rabbia e problemi nell'esprimere la rabbia
N	2	ansia
N	3	depressione
N	4	rifiuto
N	5	frustrazione
N	6	paure irrealistiche
N	7	senso di colpa
N	8	solitudine
N	9	autocommiserazione
N	10	vergogna
N	11	risentimento verso gli altri
N	12	lutti e cordogli irrisolti
N	13	senso di inadeguatezza e insicurezza
N	14	altro
**Quali emozioni positive ti porterebbero ad una ricaduta?**		
P	1	sentirmi cercato dalla vittima
P	2	eccessiva sicurezza nei confronti di un totale recupero
P	3	sentirmi compreso dalla vittima
P	4	sentimenti di affetto e vicinanza
P	5	senso di intimità
P	6	virilità o mascolinità
P	7	grandiosità e onnipotenza

P	8	senso di controllo
P	9	sollievo dalla tensione fisica ed emotiva
P	10	lasciarmi andare a fantasie sessuali o il fare sesso
P	11	altro

Quali pensieri o azioni ti porterebbero ad una ricaduta o non ti permetterebbero di interrompere il comportamento?

PE	1	razionalizzazione (il mio comportamento è giusto)
PE	2	giustificazione (non potevo e non posso fare altro)
PE	3	non ho il controllo del mio comportamento
PE	4	non sono capace di reagire allo stress
PE	5	non ho abbastanza conoscenza sulla sessualità
PE	6	non riesco ad aspettare che la terapia faccia effetto
PE	7	la mia vita non ha significato
PE	8	il crimine o il reato che ho commesso è il mio pensiero fisso
PE	9	ho fantasie, sogni e pensieri sul reato
PE	10	pensare al reato mi fa stare meglio
PE	11	non penso di aver commesso un reato
PE	12	assumere alcool e droghe
PE	13	non avere rapporti sessuali
PE	14	il dare una falsa impressione agli altri
PE	15	il pensare di essere diverso dagli altri
PE	16	il non credere di poter ricadere nel comportamento
PE	17	il pensare "non lo farò più"
PE	18	altro

Quali caratteristiche dell'ambiente in cui stai ti porterebbero ad una ricaduta o non ti permettono di interrompere il comportamento? (fai riferimento alla condizione precedente all'arresto)

E	1	presenza di potenziali vittime
E	2	accesso alle armi
E	3	accesso a droga e/o alcool conflitti
E	4	coniugali o di coppia
E	5	conflitti interpersonali
E	6	Isolamento e ritiro sociale
E	7	essere coinvolto in azioni criminali
E	8	avere contatti con autori di reati sessuali
E	9	scarse capacità sociali
E	10	incapacità di definirmi eterosessuale o omosessuale
E	11	altro

Quali motivazioni potrebbero incidere negativamente nella riuscita di un'eventuale trattamento(fai riferimento alla condizione attuale)

T	1	non partecipare alle sedute di trattamento
T	2	l'incapacità di essere sincero con il terapeuta
T	3	l'incapacità di farsi coinvolgere in gruppi di supporto
T	4	difficoltà a fidarsi degli altri in gruppi di supporto
T	5	mancata informazione sui reati sessuali
T	6	non riuscire a riconoscere le condizioni che mi porterebbero a ricadere nel reato
T	7	non riuscire ad applicare i consigli della terapia
T	8	non riuscire ad avere fiducia nel trattamento
T	9	altro

Quali altre situazioni o accadimenti potrebbero modificare il tuo comportamento? (fai riferimento alla condizione attuale)

A	1	successo nel lavoro

A	2	non sapere come impiegare il tempo libero
A	3	dare troppa importanza ai miei desideri sessuali
A	4	sentirmi di buon umore e pensare al futuro
A	5	riuscire a tenere a bada il mio desiderio sessuale
A	6	evitare di esprimere i miei sentimenti
A	7	non riuscire a parlare del reato
A	8	altro

Attachment Style Questionnaire

English version.

Table S4. Read the statements below and tick the number that most closely matches your degree of agreement. There are no right or wrong answers: 1 = Totally disagree 2 = Very much in disagreement 3 = Slightly disagree 4 = Slightly agree 5 = Very much agree 6 = Totally agree.

1	Overall, I am a worthwhile person	1	2	3	4	5	6
2	I am easier to get to know than most people	1	2	3	4	5	6
3	I feel confident that other people will be there with me when I need them	1	2	3	4	5	6
4	I prefer to depend on myself rather than other people	1	2	3	4	5	6
5	I prefer to keep to myself	1	2	3	4	5	6
6	To ask for help is to admit that you're a failure	1	2	3	4	5	6
7	People's worth should be judged by what they achieve	1	2	3	4	5	6
8	Achieving things is more important than building relationships	1	2	3	4	5	6
9	Doing your best is more important than getting on with others	1	2	3	4	5	6
10	If you 've got a job to do, you should do it no matter who gets hurt	1	2	3	4	5	6
11	It's important to me that others like me	1	2	3	4	5	6
12	It's important to me to avoid doing things that others won't like	1	2	3	4	5	6
13	I find it hard to make a decision unless I know what other people think	1	2	3	4	5	6
14	My relationships with others are generally superficial	1	2	3	4	5	6
15	Sometimes I think I am not good at all	1	2	3	4	5	6
16	I find it hard to trust other people	1	2	3	4	5	6
17	I find it difficult to depend on others	1	2	3	4	5	6
18	I find that others are reluctant to get as close as I would like	1	2	3	4	5	6
19	I find it relatively easy to get close to other people	1	2	3	4	5	6
20	I find easy to trust others	1	2	3	4	5	6
21	I feel comfortable depending on other people	1	2	3	4	5	6
22	I worry that others won't care about me as much as I care about them	1	2	3	4	5	6
23	I worry about people getting too close	1	2	3	4	5	6
24	I worry that I won't measure up to other people	1	2	3	4	5	6
25	I have mixed feelings about being close to others	1	2	3	4	5	6
26	While I want to get close to others, I feel uneasy about it	1	2	3	4	5	6
27	I wonder why people would want to be involved with me	1	2	3	4	5	6
28	It's very important to me to have a close relationship	1	2	3	4	5	6
29	I worry a lot about my relationships	1	2	3	4	5	6
30	I wonder how I would cope without someone to love me	1	2	3	4	5	6
31	I feel confident about relating to others	1	2	3	4	5	6
32	I often feel left out or alone	1	2	3	4	5	6
33	I often worry that I do not really fit with other people	1	2	3	4	5	6
34	Other people have their own problems, so I don't bother them with mine	1	2	3	4	5	6
35	When I talk over my problems with others, I generally feel ashamed or foolish	1	2	3	4	5	6
36	I am too busy with other activities to put much time into relationships	1	2	3	4	5	6

37	If something is bothering me, others are generally aware and concerned	1	2	3	4	5	6
38	I am confident that other people will like and respect me	1	2	3	4	5	6
39	I get frustrated when others are not available when I need them	1	2	3	4	5	6
40	Other people often disappoint me	1	2	3	4	5	6
